# Optical phased array receiver with mode diversity and coherent combination

**DOI:** 10.1515/nanoph-2025-0038

**Published:** 2025-03-19

**Authors:** Enge Zhang, Lei Zhang

**Affiliations:** State Key Laboratory of Information Photonics and Optical Communications & School of Integrated Circuits, 12472 Beijing University of Posts and Telecommunications, Beijing 100876, China

**Keywords:** silicon photonics, optical phased array, multimode

## Abstract

Optical phased arrays (OPAs) hold significant promise for low-cost, solid-state beam steering in LiDAR and free-space optical (FSO) communications. The field of view (FOV) is one of the key performance metrics in OPA for both optical beam transmitting (Tx) and receiving (Rx). Currently, people tend to use the same design for both the Tx and Rx parts under the hypothesis of reciprocity. In fact, Tx antennas typically generate well-controlled near-field profiles, whereas Rx apertures encounter randomly distributed incident waves due to uncontrolled reflection and propagation. This work demonstrates that leveraging mode diversity can effectively expand the FOV and enhance the receiving efficiency of Rx OPAs, irrespective of the antenna type. To efficiently utilize collected photons for coherent detection in LiDAR and FSO systems, we introduce an inversely designed mode splitter-converter and a coherent combination architecture. Unlike traditional methods, our approach effectively handles beams with varying amplitudes. As proof of concept, we designed and fabricated an 8-channel edge-emitting OPA receiver operating in TE_0_ and TE_1_ modes, employing a sparse array to suppress grating lobes within the ±90° range. Experimental results reveal an FOV of 133° for our multimode receiver, surpassing the 49° FOV of a single-mode counterpart with the same antenna array. Our approach, encompassing both mode diversity and coherent combination, introduces a new degree of freedom – higher-order spatial modes – with the potential to significantly advance OPA receiver design.

## 1 Introduction

Optical phased array (OPA) has emerged as a solid-state beam steering solution that enables fast and accurate beam scanning through precise phase control of antenna elements in an array [1], [2], [3], [4], [5], [6], [7], [8], [9], [10], [11], [12], [13], [14], [15], [16], [17], [18], [19], [20]. The OPA technology can find applications in light detection and ranging (LiDAR) for ranging and detection [1] and free-space optical communication (FSO) for acquisition, tracking, and pointing (ATP) [2], [3]. The OPA approach eliminates the need for mechanical scanning components, thereby improving the stability and reliability of the systems [4], [5], [6], [7]. The use of silicon-based materials and CMOS-compatible fabrication techniques enables the fabrication of low-cost and large-scale optical phased arrays [8], [9], [10], [11], [12]. This is beneficial for miniaturizing, lightweighting, and reducing the divergence angle of the OPA to adapt to different scenarios in LiDAR and FSO systems.

**Figure 1:**
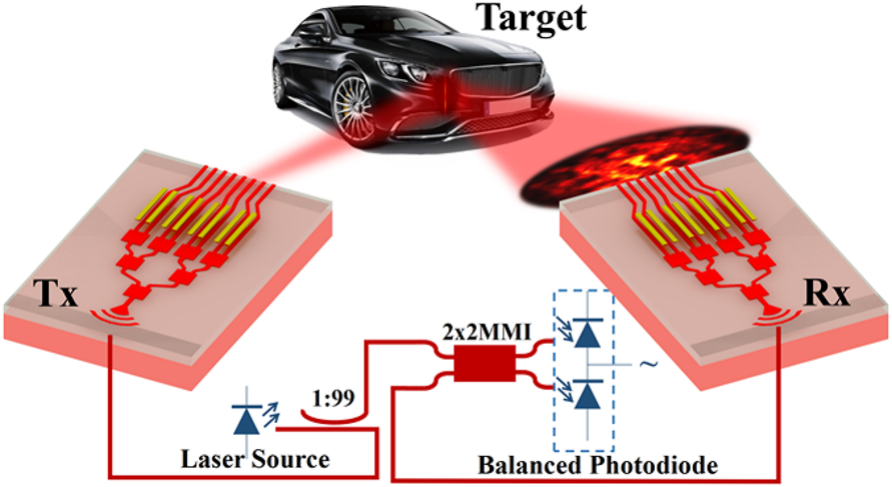
Schematic of the transmitting and receiving process of a LiDAR system with optical phased array (OPA) and coherent detection.

As shown in Figure 1, the transmitting (Tx) and receiving (Rx) processes in a LiDAR system with OPA are not simply reciprocal. For the Tx OPA, we adjust the amplitude and phase of the optical wave in each channel with certain polarization and spatial mode [2], [3], [4], [5], [6], [7], [8], [9]. For the Rx OPA, however, the received optical wave incident on the antenna aperture has a random spatial distribution (i.e., a dynamic composition of orthogonal modes with fluctuating amplitudes and phases) due to unpredictable reflector surface roughness and atmospheric fluctuations. Therefore, using the Tx OPA configuration for the Rx OPA may not ensure optimal receiving efficiency.

Currently, most LiDAR systems based on OPAs primarily use single-mode systems [5]. There has been some work to explore the potential of higher order modes in OPAs to improve the LiDAR performance [21], [22]. K. Wang et al. proposed a Tx OPA using TE_0_ and TE_1_ modes for parallel beam steering [21]. R. Fatemi et al. proposed a multimode antenna using an inverse design method resulting in a large FOV [22]. These theoretical studies show that it is promising to use higher order modes in OPAs. However, these works lack a general and in-depth analysis of the use of higher order modes for the Rx OPA. For example, they use specific antennas and lack the discussion of the beam combination solution after the photons have been collected into the chip, which is essential to fully exploit the multimode receiving scheme.

In this paper, we present a comprehensive analysis of multimode OPA receivers. We theoretically demonstrate the advantage of multimode technology in extending the FOV of three typical antennas (i.e., end-fire facet antenna, small grating antenna, and long grating antenna) and improving the reception efficiency. To handle the received multimode beam, we use an inverse design method to develop a mode splitter-converter that exhibits an insertion loss of less than 1 dB and crosstalk of less than −22.5 dB in C-band. In addition, we present a coherent combination architecture that is well suited to handling beams with different amplitudes. As a proof of principle, we have designed an 8-channel end-emitting OPA receiver on silicon-on-insulator (SOI) substrate using TE_0_ and TE_1_ and experimentally verified its effectiveness in extending the receiving FOV.

The paper is organized as follows. In Section 2, we introduce the constituent parts of the OPA receiver. In Section 3, we provide a detailed analysis of the performance improvement of three typical antenna elements with higher order modes, as well as the design of one-dimensional 8-, 32- and 128-channel sparse arrays. In Section 4, we introduce the inverse design of the mode splitter-converter. In Section 5, we present the design of the coherent combination architecture. In Section 6, we present the simulation and characterization of an 8-channel dual-mode OPA chip. In Section 7, we summarize the work and present our perspective on future research.

## 2 OPA receiver architecture

**Figure 2:**
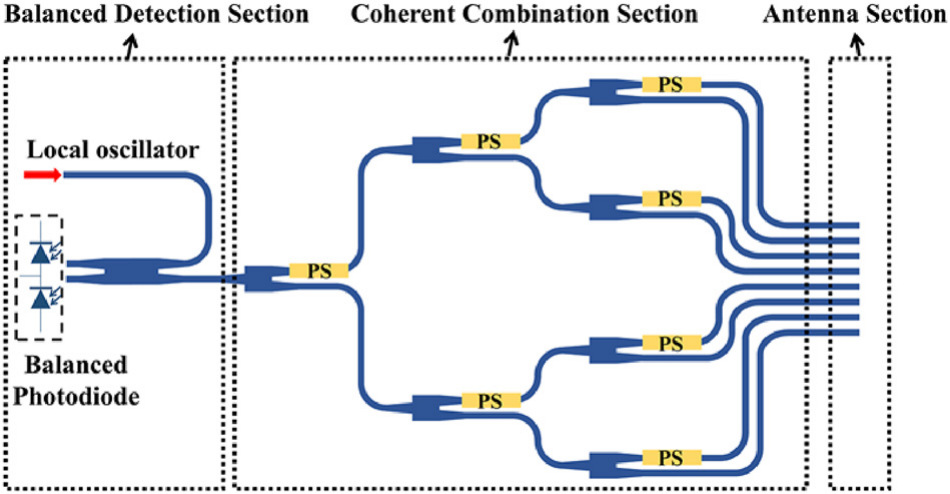
Schematic of OPA receiver with functional blocks.

The OPA receiver can be divided into three sections, namely the antenna, the coherent combination section, and the balanced detection, as shown in Figure 2. The light reflected from the target is collected by the antenna section, which typically has multiple ports. The coherent combination section combines the light from these ports into one single-mode beam and delivers it to the balanced detection section to interfere with the local oscillator beam in a 2 × 2 optical coupler. Mode splitter-converters must be incorporated before the coherent combination section because we cannot make the orthogonal spatial modes interfere with each other to achieve the coherent combination.

There are three key factors in the design of the Rx OPA, namely the FOV, optical reception efficiency, and coherent combining efficiency. The first factor determines the angular range within which the system can effectively capture incoming photons. The second factor refers to the antenna element’s efficiency to capture light into the chip. The third factor focuses on the degree to which the light collected by the antenna is effectively combined into one single-mode beam. The first two factors are closely related to the antenna section (both the antenna element and the array arrangement) in Figure 2. The last factor is related to the coherent combination structure. With these considerations in mind, we will now take a closer look at the antenna section and the coherent combination architecture, as well as the mode splitter-converter in between.

## 3 Antenna section of OPA

The antenna section is required to have large FOV and high receiving efficiency. The far field pattern of the antenna array can be described as follows [23].

(1) $$E_{\mathrm{far}}^{\mathrm{array}}\left(\theta ,\varphi \right)=E_{\mathrm{far}}^{\mathrm{element}}\left(\theta ,\varphi \right)\cdot A\left(\theta ,\varphi \right)$$

where $E_{\mathrm{far}}^{\mathrm{array}}$ is the far field of the antenna array, $E_{\mathrm{far}}^{\mathrm{element}}$ is the far field of the element, and *A* is the array factor, which is only determined by the array arrangement.

It can be seen from Equation (1) that the OPA FOV is determined by both the far field of the element and the array factor. The antenna element far field profile set the upper limit for the OPA FOV. It may be further limited by the array factor due to the grating lobes, such as in uniform array with element pitch over half wavelength. To distinguish between them, we designate the first as element-limited FOV and the second as array-limited FOV. The smaller of these two determines the overall FOV of the system.

**Figure 3:**
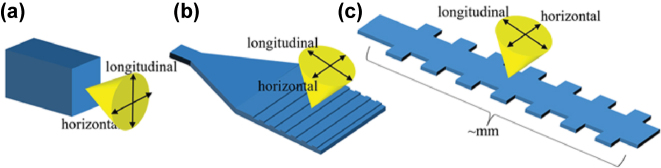
The schematic diagram of the three major antenna elements in OPA, which are (a) end-fire facet antenna, (b) small grating antenna, and (c) long grating antenna.

We initiate our discussion with the antenna elements. As shown in Figure 3, three types of antenna elements are utilized in OPA, namely the end-fire facet antenna, the small grating antenna, and the long grating antenna. Typically, the first two types of antennas operate at a single wavelength. Utilizing an end-fire facet antenna alone allows for one-dimensional (1D) scanning. By arranging the small grating antennas in a two-dimensional (2D) array configuration, 2D scanning can be accomplished. The long grating antennas are typically on the order of millimeters, which can achieve 2D scanning by manipulating the phase distribution among the antennas for one direction and altering the wavelengths for the other direction.

The utilization of multiple wavelengths in the long grating antenna distinguishes it from other types of antennas. In the case of long grating antennas, scanning in the longitudinal direction is accomplished through variations in wavelength. To attain high pointing accuracy in this direction, it is imperative that the far-field pattern of the antenna element along this axis be as narrow as possible. Conversely, for the end-fire facet antenna and the small grating antenna, achieving a large system FOV necessitates that the far-field patterns along both axes be as broad as possible. Below is a detailed analysis of these three types of antennas.

### 3.1 End-fire facet antenna

**Figure 4:**
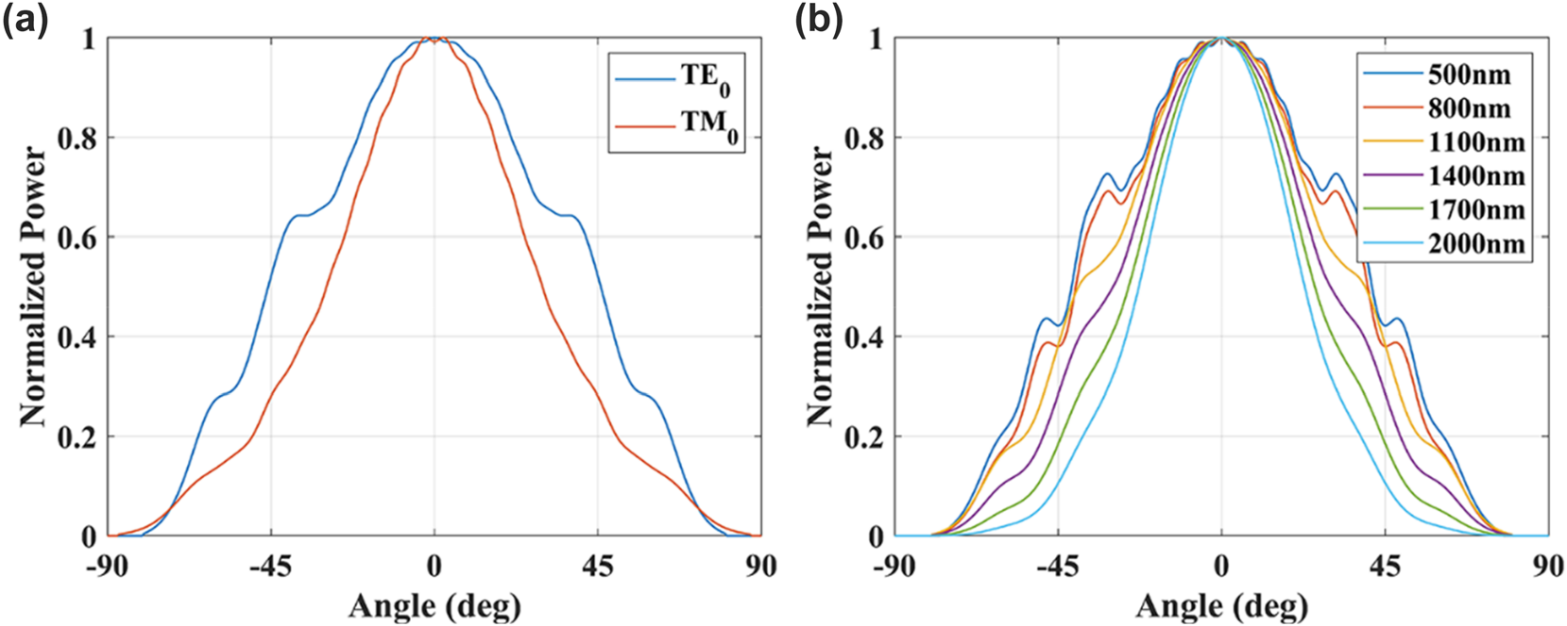
Simulated far-field patterns of the waveguide. (a) TE_0_ and TM_0_ modes, 500 nm width. (b) TE_0_ mode, varying widths.

The angular range of the LiDAR receiving system is limited by the antenna element. Therefore, it is desirable to have a receiving range of the antenna element that is as large as possible to support reception over a sufficiently large angular range. Typically, the antenna of the edge-emitting system operates in fundamental mode, which allows for a relatively narrow waveguide width. The far-field patterns of the TE_0_ and TM_0_ modes have been simulated and are shown in Figure 4a. The angular ranges exhibited by the two modes are comparable, which cannot effectively increase the receiving range of the LiDAR system. Therefore, using the TE_0_ mode is sufficient for sake of extending the FOV.

If we want to investigate the far-field pattern of higher-order modes, it becomes necessary to increase the width of the waveguide. It is important to note, however, that this change will inevitably affect the far-field pattern of the TE_0_ mode as well. We have performed simulations of the far-field patterns of the TE_0_ mode of the waveguide with varying widths, and the results are shown in Figure 4b.

As shown in Figure 4b, the variation of the waveguide width has a negligible effect on the far-field pattern of the TE_0_ mode. A change in the width from 500 nm to 2,000 nm is accompanied by a corresponding change in the full width at half maximum (FWHM) of the far-field pattern from 82.6° to 77.8° (∼5.7 %). Consequently, the change in the far-field pattern of the TE_0_ mode due to changes in the waveguide width could be negligible.

**Figure 5:**
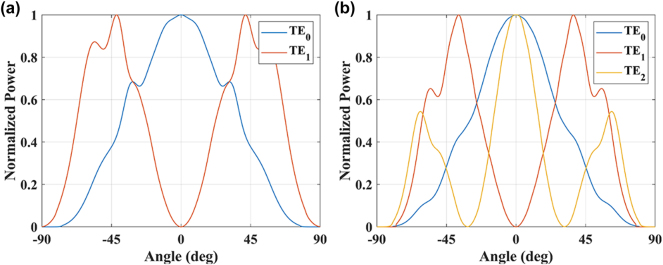
The far field patterns of (a) TE_0_ and TE_1_ modes for a 900 nm SOI waveguide and (b) TE_0_, TE_1_, and TE_2_ modes for a 1,400 nm SOI waveguide.

Next, the far-field patterns of TE_0_ and TE_1_ modes for a 900-nm waveguide and the far-field patterns of TE_0_, TE_1_, and TE_2_ modes for a 1,400-nm waveguide have been simulated, and the results are shown in Figure 5. It can be seen that the use of multimode effectively improves the FOV of the element. For example, at 45°, all modes are able to receive light, and the reception efficiency of the antenna is indeed improved compared to the use of single mode. It also can be seen that the far-field patterns of TE_0_ and TE_1_ modes are complementary to each other, while the far-field pattern of TE_2_ mode is almost included in the far-field patterns of the first two modes.

It should be pointed out that, the employment of TM_0_ and TE_2_ is beneficial for collecting photons in different polarization and spatial modes, which will definitely improve the receiving efficiency. But we need more complex circuits for mode conversion and way more channels for coherent combination. Thus, in the following chip design, we only utilize the TE_0_ and TE_1_ modes.

### 3.2 Small grating antenna

**Figure 6:**
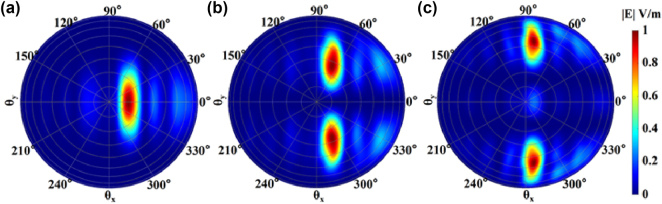
The far field patterns of (a) TE_0_, (b) TE_1_, and (c) TE_2_ modes of the small grating antenna.

To ensure the large far-field pattern of the antenna, the size of the grating is as small as possible. Here, we have designed a 4 μm × 2 μm grating antenna, the duty cycle is 0.7, and the period is 0.63 μm. The far-field patterns of the TE_0_, TE_1_, and TE_2_ modes are shown in Figure 6. From the figure, we can see that the use of multimode does indeed improve the FOV of the antenna element and receiving efficiency of certain degree. The emitting angle at the longitudinal axis is determined by the effective refractive index (*n*_eff_). Since the three modes have different *n*_eff_, the emitting angles of the three modes are slightly different as shown in Figure 6. Specifically, the FOV of the TE_0_ mode is 11.55° × 34.91°. The FOV for the three-mode system is 19.59° × 124.26°. Thus, by adopting all three modes instead of just TE_0_ mode, the FOV increases approximately 1.69 times in the longitudinal direction and approximately 3.56 times in the horizontal direction.

### 3.3 Long grating antenna

**Figure 7:**
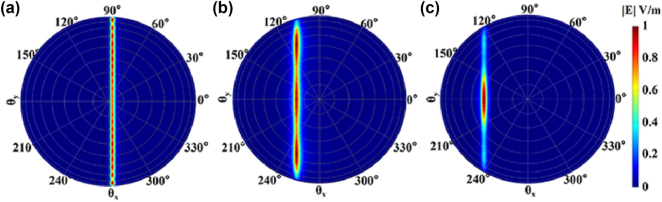
The far field patterns of (a) TE_0_, (b) TE_1_, and (c) TE_2_ modes of the long grating antenna.

**Figure 8:**
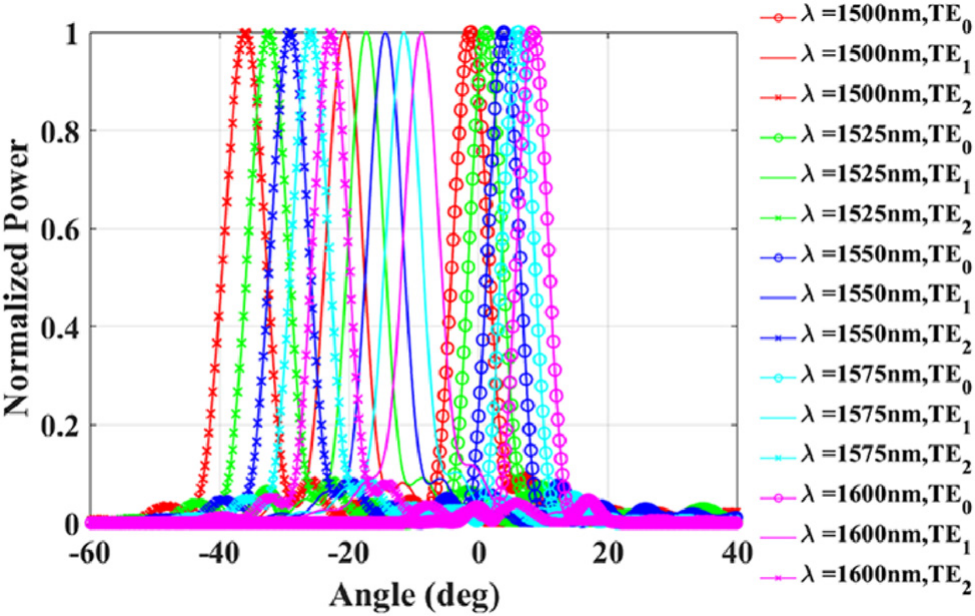
Far field in longitudinal axis of TE_0_, TE_1_, and TE_2_ modes with varying wavelength.

A long grating antenna achieves horizontal scanning by utilizing the phase manipulation of its elements. At a specific wavelength, the antenna element FOV in the horizontal direction is required to be large to cover enough angular range. To support this wide FOV, the width of each antenna element is typically narrow, around 500 nm [15], [16]. However, if multimode is desired, the waveguide width must be increased, which may lead to a concern about a reduced horizontal FOV. Here, we have designed a long grating antenna element with a width of 1.4 μm, a period of 1 μm, and a duty cycle of 0.7. The far field patterns of the multimode are shown in Figure 7. It can be seen from Figure 7a that even if the width is wider, it is still possible to get large FOV in the horizontal direction. However, it should be noted that for higher-order modes, the horizontal FOV will decrease. Specifically, the horizontal FOV for TE_0_, TE_1_, and TE_2_ modes is 166.49°, 121.38°, and 34.95°, respectively. From Figure 7b and c, it is clear that TE_1_ and TE_2_ have different pointing angles in the longitudinal direction. We have also simulated the far field of the longitudinal axis while varying the wavelength, as depicted in Figure 8. Different modes are represented by different symbols, with the TE_0_ labeled with circles. Different wavelengths are represented by different line colors. From this figure, it can be observed that the total FOV is approximately 3 times larger than when only the single mode is used.

### 3.4 Antenna array arrangement

To improve the far-field scanning range of the LiDAR system, we use the self-adapted particle swarm optimization (SAPSO) algorithm to optimize the array arrangement [24]. The formulas of SAPSO are presented below.

(2) $$\begin{array}{ll} v^{i}\left(n+1\right) & =\omega v^{i}\left(n\right)+c_{1}r_{1}\left(p_{\mathrm{best}}^{i}-x^{i}\left(n\right)\right)\\ & \;+c_{2}r_{2}\left(g_{\mathrm{best}}-x^{i}\left(n\right)\right)+Gr \end{array}$$

(3) $$x^{i}\left(n+1\right)=\alpha g_{\mathrm{best}}+\left(1-\alpha \right)x^{i}\left(n\right)+v^{i}\left(n+1\right)$$

(4) $$\omega =\left\{\begin{array}{c} \omega_{s}+\left(\omega_{e}-\omega_{s}\right)\frac{f_{g}}{f_{\mathrm{aim}}}f_{g}\leq f_{\mathrm{aim}}\;\\ 0\; \end{array}\right.$$

where *α* is the global best weight, *G* is the random factor, *r* is a random vector in which each dimension is a random number from −1 to 1, *f*_aim_ is the value we set close to our target figure of merit (FOM), *f*_*g*_ is the group optimum, *ω*_*s*_ is the start of inertia weight, and *ω*_*e*_ is the end of inertia weight, which satisfies *f*_*g*_ = *f*_aim_. The random factor *G* and global best weight *α* are defined as follows:

(5) $$G=G_{s}+\left(G_{e}-G_{s}\right)\frac{f_{g}}{f_{\mathrm{target}}}$$

(6) $$\alpha =\frac{i-1}{N-1}$$

where *f*_target_ is our target FOM, *G*_*s*_ is the start of inertia weight, *G*_*e*_ is the inertia weight designated for the iteration, which satisfies *f*_*g*_ = *f*_target_, and *N* is the total number of particles in the population.

**Figure 9:**
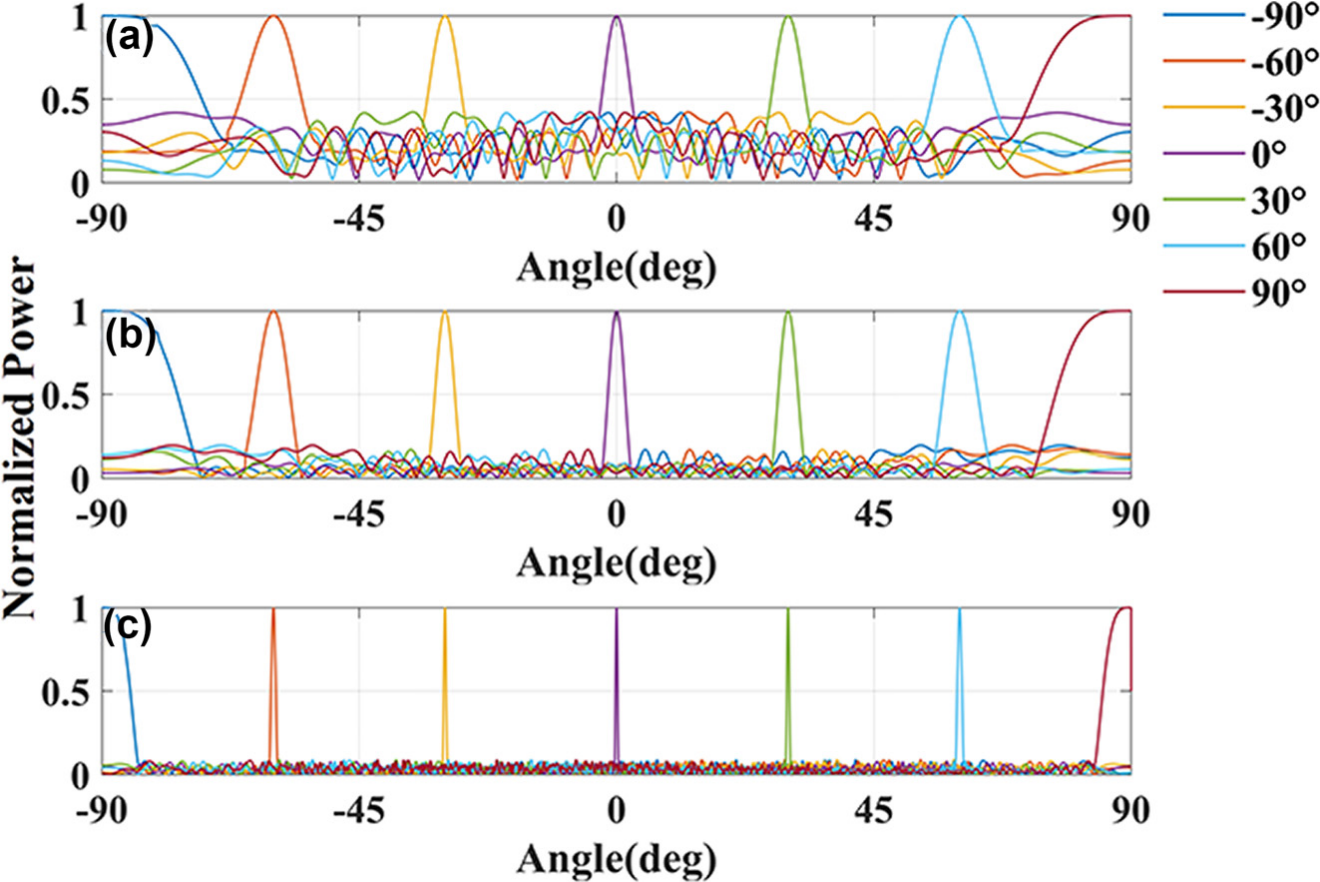
Array factors for (a) 8, (b) 32, and (c) 128 channels antenna array pointing at different angles.

We use the SAPSO algorithm to optimize the position of the elements in the array, with the figure-of-merit (FOM) being the side lobe level (SLL) in the range of ±90°. We have optimized the array factor of 8, 32, and 128 channels, as shown in Figure 9. From the figure, it can be observed that there is no grating lobe present in the range of ±90°, and the SLL is 4.6 dB, 7.12 dB, and 10.39 dB, respectively.

## 4 Mode splitter-converter

**Figure 10:**
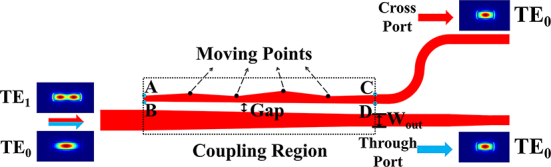
Schematic of the mode splitter-converter.

After optical energy in multimode field has been received by the antenna, we need to separate modes in different orders and convert higher modes into fundamental mode for further coherent combination. Mode splitter-converters have been studied in a few works [24], [25], [26], [27]. In 2016, C. Sun et al. employed adiabatic couplers (ACs) to create designs featuring insertion loss (IL) of less than 1 dB and crosstalk below −20 dB, despite a length of 200 μm. In 2018, R.B. Priti et al. utilized cascaded multimode interferometer (MMI) couplers, yielding devices with sizes exceeding 120 μm, a maximum IL of 7.3 dB in the C-band, and crosstalk below −20 dB. In 2021, B. Paredes et al. adopted a tapered asymmetric directional coupler (ADC) for designs measuring 75 μm, achieving IL of less than 1.2 dB, and crosstalk below −16 dB. More recently, in 2024, W. Jiang et al. employed a direct binary search algorithm to develop ultra-compact silicon mode (de)multiplexers with dimensions of 4 μm × 1.25 μm, IL below 2.16 dB, and crosstalk below −15 dB. In this work, we integrate the advantages of inverse design and ADC structures by utilizing the SAPSO algorithm in conjunction with cubic spline interpolation to design mode splitter-converters [28], with the schematic shown in Figure 10.

To support TE_0_ and TE_1_ modes, we choose the waveguide width to be 900 nm. Therefore, the input waveguide width on the left side of the structure in Figure 10 is 900 nm. When the light enters from the input waveguide and passes through the coupling region, the TE_0_ mode will be directly output from the through port. Meanwhile, the TE_1_ mode will be transformed into the TE_0_ mode and subsequently output from the cross port. The coupling region of the mode splitter-converter is 20 μm and consists of two waveguides with variable gap between them. The lower waveguide is a trapezoid with a variable width *W*_out_ at the end. The upper waveguide is formed by four points A, B, C, D and some movable points in the middle, where the curve on the upper side is obtained through cubic spline interpolation from points A, C, and the movable points. All the points have fixed horizontal coordinate positions (uniformly distributed in the 20 μm region) and variable vertical coordinate positions. It should be noted that when *W*_out_ and gap are determined, the positions of B and D are determined correspondingly. The SAPSO algorithm is employed to optimize the aforementioned variables and identify the device structure with the lowest insertion loss. The expression of the FOM employed in the optimization is as follows.

(7) $$FOM=\left(P_{TE_{0}}^{\mathrm{Through}}-P_{TE_{0}}^{\mathrm{Cross}}\right)+\left(P_{TE_{1}}^{\mathrm{Cross}}-P_{TE_{1}}^{\mathrm{Through}}\right)$$

where $P_{TE_{0}}^{\mathrm{Through}}$ and $P_{TE_{0}}^{\mathrm{Cross}}$ are the output powers of the through port and cross port, respectively, under TE_0_ mode input conditions, $P_{TE_{1}}^{\mathrm{Through}}$ and $P_{TE_{1}}^{\mathrm{Cross}}$ are the output powers of the through port and cross port, respectively, under TE_1_ mode input conditions.

**Figure 11:**
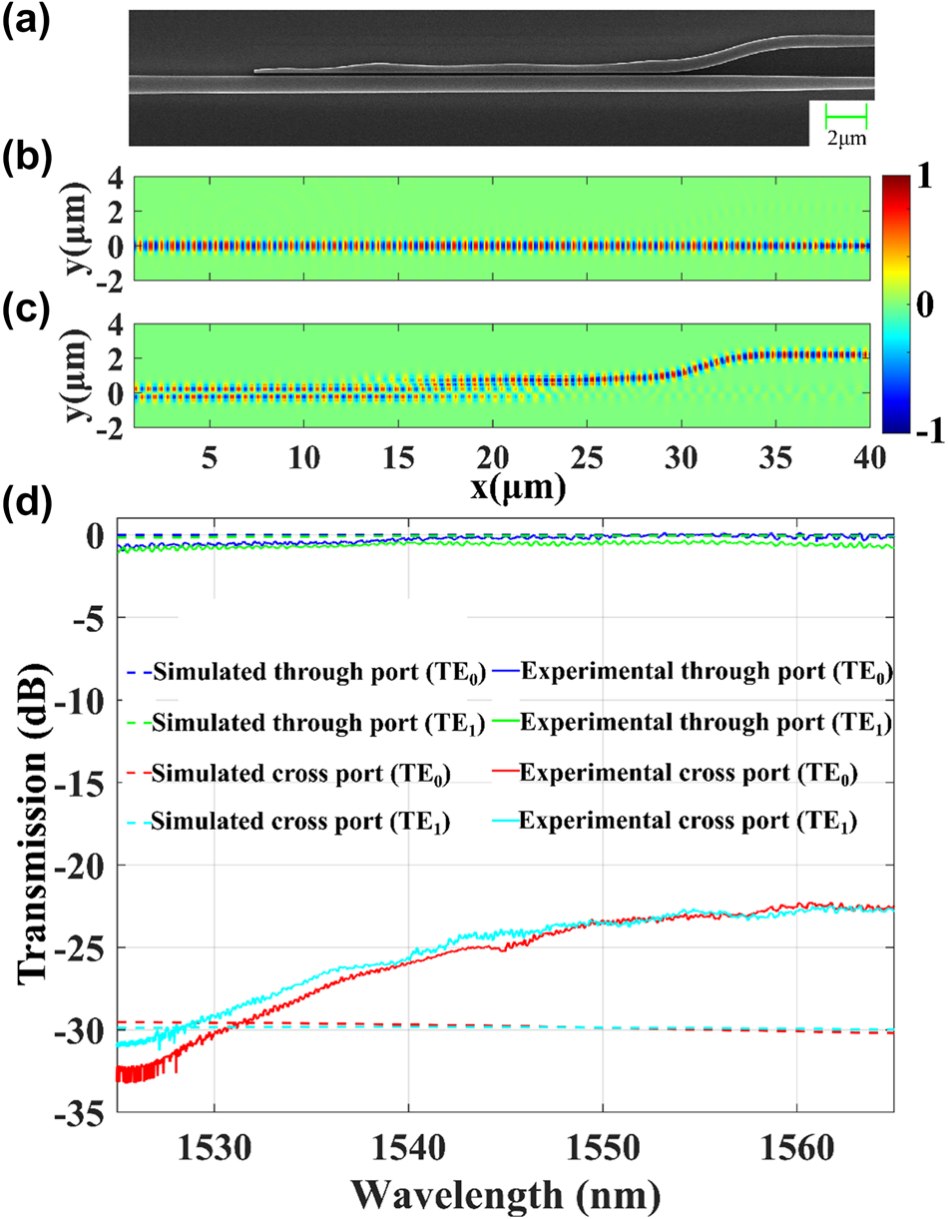
Mode splitter-converter simulation and experimental results. (a) The scanning electron microscope (SEM) image. (b) and (c) The simulated electric-field distribution (*E*_y_) with TE_0_ mode input and TE_1_ mode input, respectively. (d) C-band transmission characteristics.

During the actual optimization process, 10 points have been utilized. The simulation and experimental results of the final optimized structure are shown in Figure 11. It can be seen that the experimental (simulated) maximum loss of the TE_0_ and TE_1_ modes is 0.96 dB (0.16 dB) over the C-band, and the maximum crosstalk is −22.5 dB (−29.8 dB).

During the actual optimization process, 10 points have been utilized. The simulation and experimental results of the final optimized structure are shown in Figure 11. It can be seen that the experimental (simulated) maximum loss of the TE_0_ and TE_1_ modes is 0.96 dB (0.16 dB) over the C-band, and the maximum crosstalk is −22.5 dB (−29.8 dB). Table 1 presents a comparative analysis of the proposed mode splitter-converter with previously reported designs.

**Table 1:** Comparison with other structures for mode splitter-converter.

Ref.	Structure	Length (μm)	IL (dB)	Crosstalk (dB)
[24]	Adiabatic couplers	200	∼1	<−20
[25]	Cascaded MMI	>120	7.3	<−20
[26]	Asymmetric directional coupler	75	1.2	<−16
[27]	Inverse-designed adiabatic coupler	4	2.16	<−15
This work	Inverse-designed asymmetric directional coupler	20	0.96	<−22.5

## 5 Coherent combination structure

**Figure 12:**
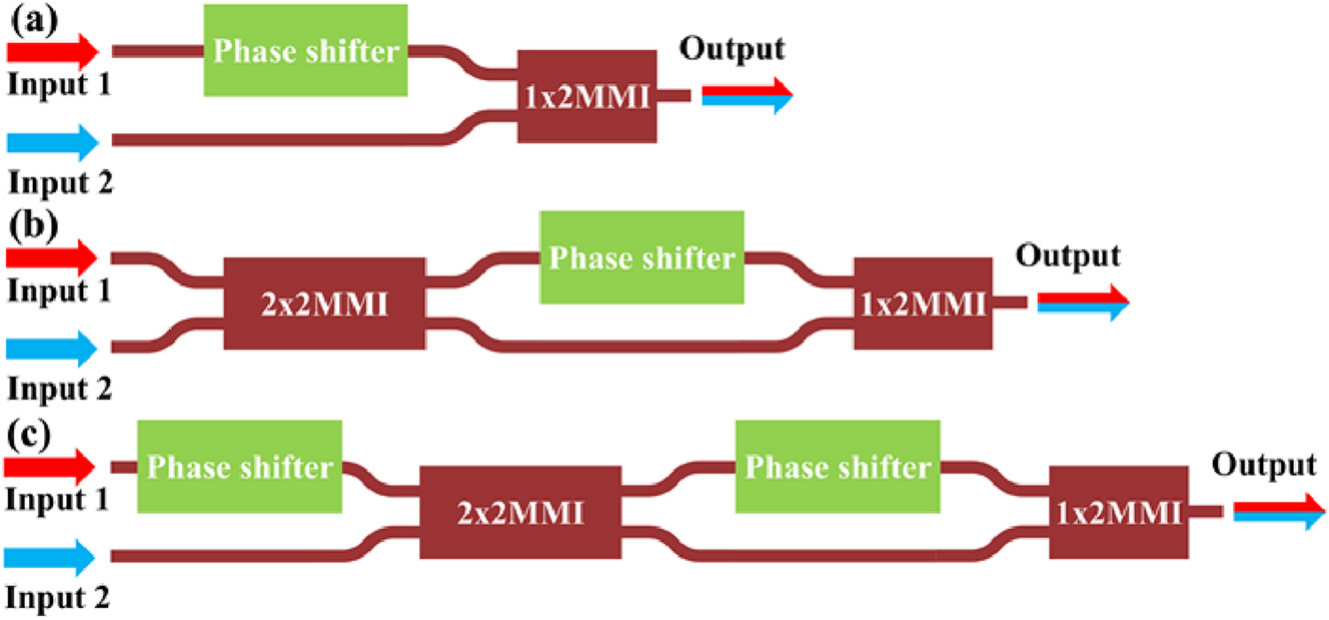
Optical combining structures. (a) Traditional design (TCCS). (b) Hybrid design (HCCS). (c) Fully coherent combining structure (FCCS).

Once the TE_1_ mode has been converted to TE_0_ mode, the subsequent step is to coherently combine light in all paths. Figure 12a shows the traditional coherent combining structure (TCCS), which consists of a phase shifter and a 1 × 2 MMI. As illustrated in Figure 5, when the reception angle is 0°, the TE_0_ mode of the antenna exhibits the highest reception efficiency, while the TE_1_ mode shows the lowest reception efficiency. For such cases, the TE_0_ and TE_1_ modes have obviously different field amplitudes. Using TCCS to handle such cases will result in intrinsic 3 dB loss. In other words, the output is half of the higher input.

**Figure 13:**
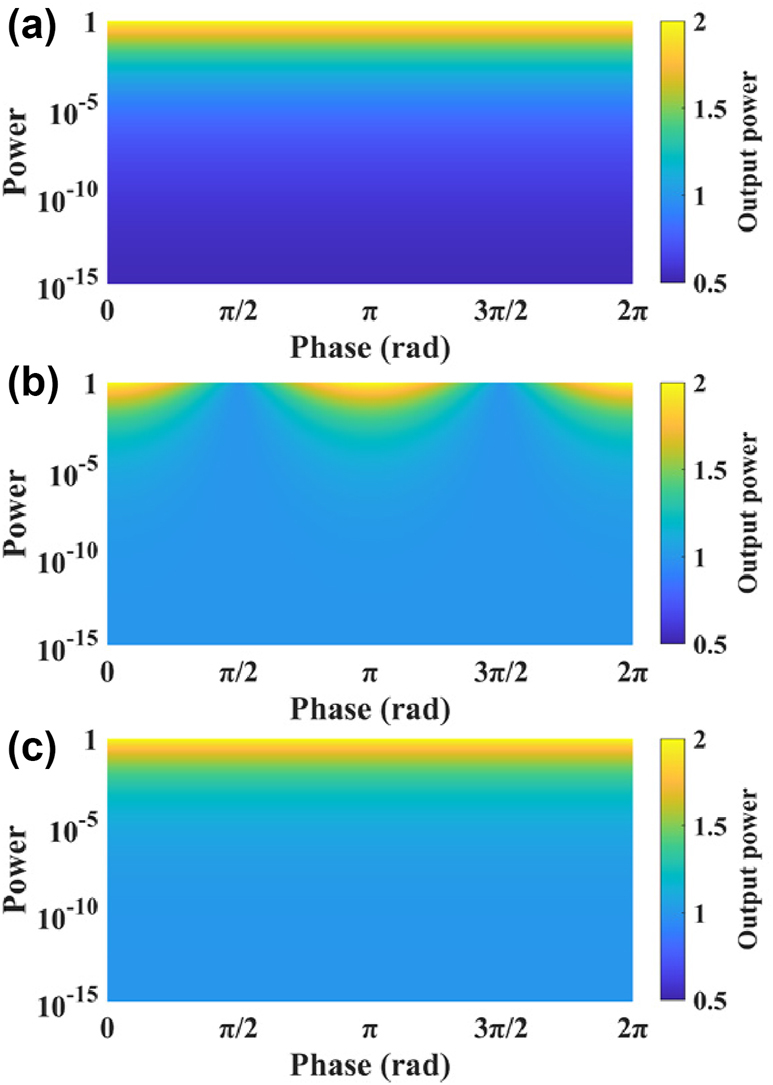
Optical output power of (a) TCCS, (b) HCCS, and (c) FCCS with one input fixed and the other one varying in power and phase.

To better illustrate the performance of the TCCS, we conduct the following simulation. The optical power of input 1 is fixed to be 1, while the phase is set to be 0. The optical power and phase of input 2 are varied, and the maximum output power is calculated. The results are shown in Figure 13a. When the optical power of Input 2 is very low, the output power of TCCS is close to 0.5, i.e., half of the optical power of Input 1.

To address this issue, we propose the hybrid coherent combining structure (HCCS) as shown in Figure 12b. It adds one 2 × 2 MMI into the TCCS to homogenize the two inputs to the two arms. This can partially solve the problem with highly imbalanced inputs in TCCS. The coherent combination effect is also influenced by the relative phase relationship between the two inputs, which will be discussed later. We conduct the same simulation for the HCCS, with the result shown in Figure 13b. When the optical power of Input 2 is very low, the output power of HCCS is close to 1, i.e., there is no loss at all.

The architecture shown in Figure 12c can have perfect coherent combining with two phase shifters, which is proposed by D. Miller [29], [30], [31], [32], [33]. We call it fully coherent combining structure (FCCS) afterward. This structure can combine two inputs with arbitrary amplitudes and phase relationships (Figure 13c). The number of phase shifters in the FCCS is two times more than the HCCS. For practical OPAs with thousands of antennas, this will result in heavy burden in hardware to control and algorithm to find the optimized setting. Thus, we choose the HCCS as a balanced solution in our following design.

**Figure 14:**
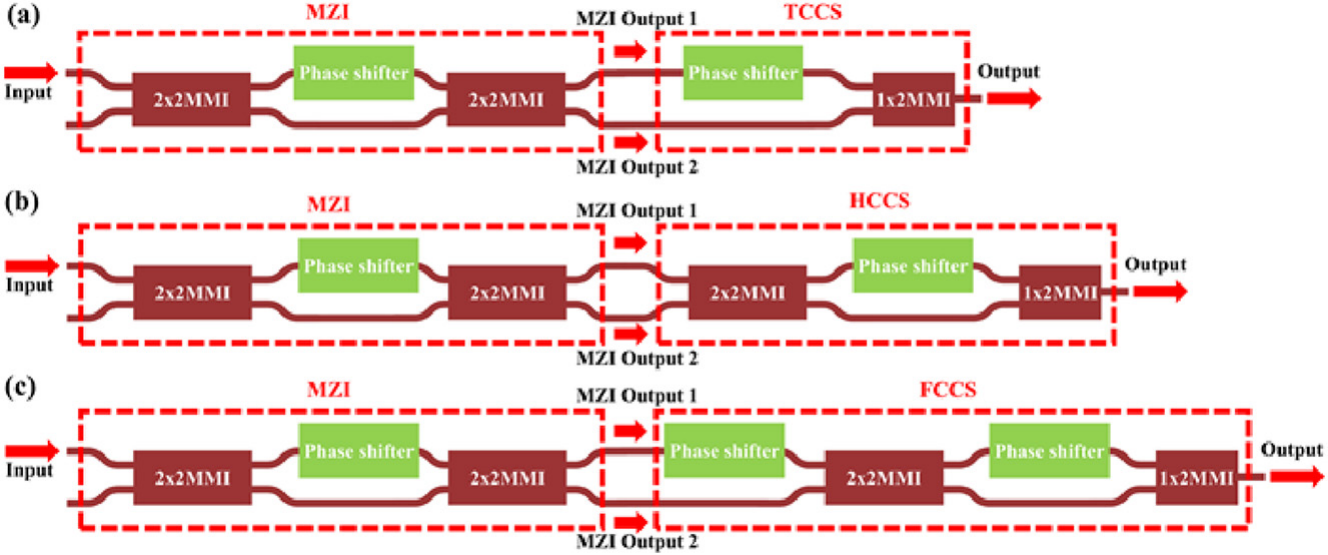
The test structure of (a) TCCS, (b) HCCS, and (c) FCCS.

To further illustrate the above analysis, we use a Mach–Zehnder interferometer (MZI) to function as a variable optical splitter (Figure 14) to provide two inputs for the three structures in Figure 12.

**Figure 15:**
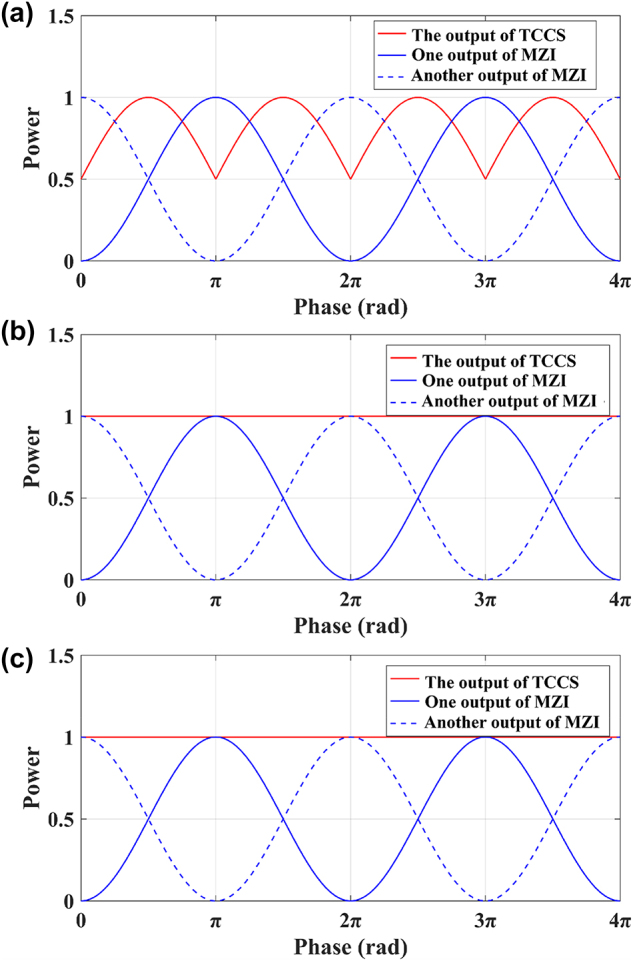
The output of (a) TCCS, (b) HCCS, and (c) FCCS connected to the MZI as a function of the phase difference between the two arms of the MZI.

We simulated the structures in Figure 14 while setting the input to 1 and observe the variation of the output as a function of the phase difference between the two arms of the MZI, as shown in Figure 15.

As shown in Figure 15a, changes in the phase difference between the two arms of the MZI result in corresponding changes in the power of its dual outputs, which in turn affects the output of the TCCS. However, the HCCS consistently combines all the light, unaffected by changes in the MZI outputs. This is due to the fact that the phase difference between the two outputs of the MZI is always 0 or *π*. As shown in Figure 13b, the HCCS has the optimal combining effect at this point, resulting in complete light combination. In contrast, the FCCS can have optimal combining effect for arbitrary amplitudes and phase relationships of the two inputs (Figure 15c), which is not chosen by us to simplify the hardware and algorithm.

**Figure 16:**
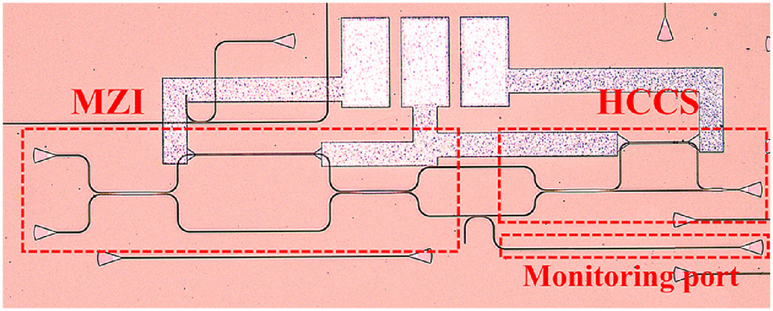
The microscope photograph of HCCS test structure.

**Figure 17:**
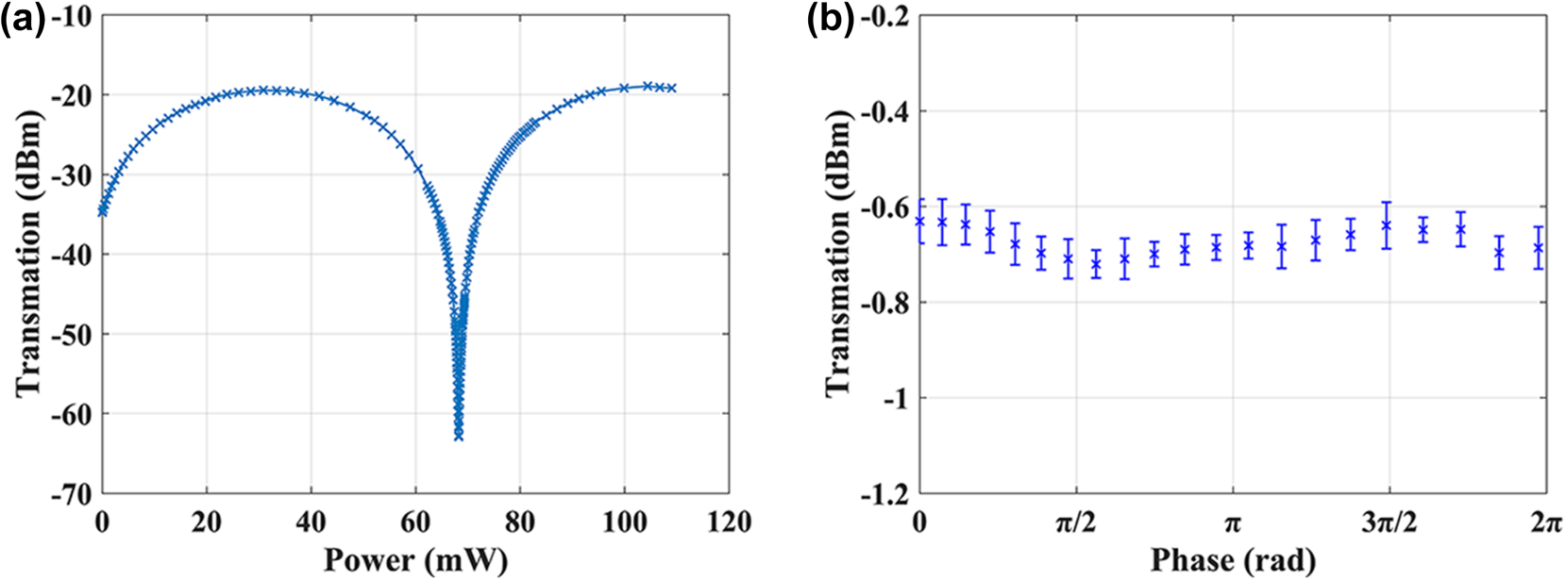
Characterization of the HCCS test structure (as shown in Figure 16). (a) Transmission of the MZI lower port during phase shifter tuning. (b) Measured performance of the HCCS test structure.

Since we have chosen the HCCS, we verify its function with experiment using the structure in Figure 14b. The microscope image of the test structure is shown in Figure 16. In order to determine the variation range of the splitting ratio of the MZI, 1 % of the light from the lower port of the MZI is tapped with a directional coupler for monitoring. The experimental results are shown in Figure 17.

Figure 17a shows the spectrum from the monitoring port. The extinction ratio of the MZI is 43.46 dB, which provides a wide dynamic range for the subsequent characterization of the light combining capability of the HCCS. Figure 17b shows that the measured output power of HCCS remains constant regardless of the phase different between the two arms in MZI, which shows a consistent behavior consistent with the simulation result (Figure 15b).

## 6 Experimental results of OPA receiver

**Figure 18:**
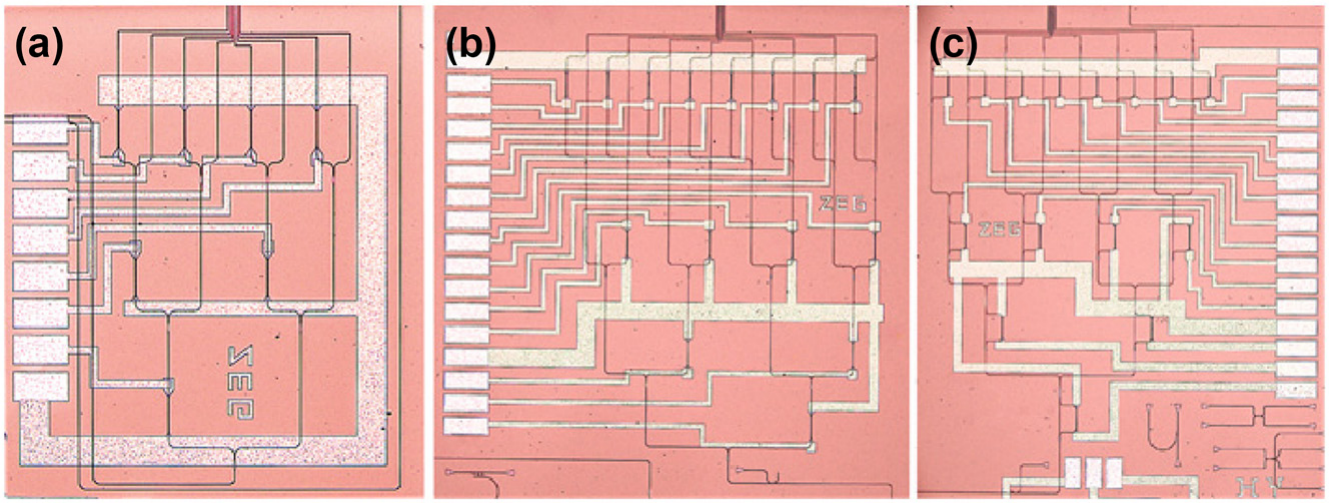
Microscope images of (a) the first scheme, using TE_0_ mode reception with TCCS for coherent combination, (b) the second scheme, utilizing TE_0_ and TE_1_ dual-mode reception with TCCS for coherent combination, and (c) the third scheme, adopting TE_0_ and TE_1_ dual-mode reception but with HCCS for coherent combination.

To demonstrate the effectiveness of the proposed multimode OPA receiving scheme, three schemes are investigated (Figure 18). The first one uses only TE_0_ mode with TCCS for coherent combination (Figure 18a). The second one uses TE_0_ and TE_1_ modes and also the TCCS for coherent combination (Figure 18b). The third one uses TE_0_ and TE_1_ modes and the HCCS for coherent combination (Figure 18c). All the three schemes use the same 900 nm antenna element and the nonuniform array arrangement described in Section 3.4. There are 900-to-500 nm linear tapers between the antenna and the TCCS in Figure 18a. Thus, only the TE_0_ mode contributes to the light collection. For those in Figure 18b and c, mode splitter-converters are employed between the antenna and the coherent combining structures. Thus, photons in both the TE_0_ and TE_1_ modes are utilized.

**Figure 19:**
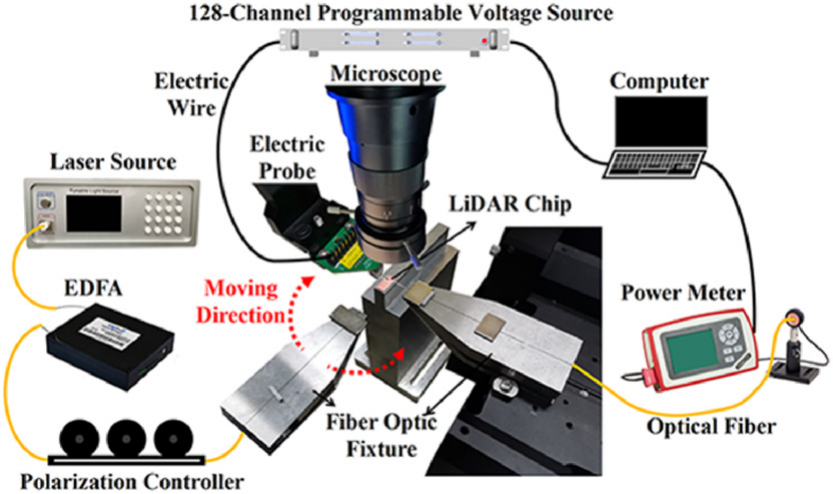
Experimental setup of the OPA chip test.

**Figure 20:**
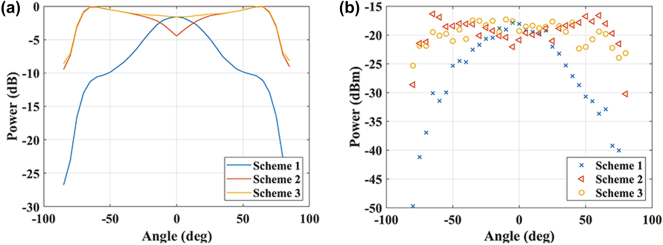
Performance of three receiving schemes. (a) Simulated data. (b) Experimental data.

The experimental setup is shown in Figure 19. Due to the small aperture of the proof-of-principle OPA chip, we direct the laser light to it through a single-mode fiber positioned 0.5 mm from the chip facet. It should be noted that when the optical fiber is obliquely incident on the OPA chip, the TE_0_ and TE_1_ modes of the antenna can be excited. We then adjust the phase shifters within the OPA to find the maximum output intensity that can be achieved. The simulation and experimental results of the three OPA chips in Figure 18 are shown in Figure 20.

As shown in Figure 20, the performance of the dual-mode scheme is clearly superior to that of the single-mode scheme. As shown in Figure 20b, Scheme 3 has an FOV of 133°, which significantly exceeds that of Scheme 1, which has an FOV of 49°. It should be pointed out that the FOV considered here is an angular range, which has power level 3 dB lower than that of the maximum. So, the FOV of Scheme 2 can be divided into two parts from −68° to −36° and from 40° to 68°, forming a total FOV of 60°.

Figure 20 shows that for angles other than near 0°, the difference between the two dual-mode receiving schemes is not significant. This is because in these angle regions, the input light stimulates both the TE_0_ and TE_1_ modes with random amplitude and phase relationship. This leads to the fluctuations in the receiving efficiencies of dual-mode schemes in Figure 20b.

We compare our results with other edge-emitting OPAs reported in literature, as shown in Table 1. Our device exhibits the largest FOV. It should be pointed out that the FOV is determined by both the antenna element and the array arrangement. We choose the smaller one as the system FOV for comparison in Table 2.

**Table 2:** Comparison with other edge-emitting OPAs.

Ref.	Antenna number	Mode number	FOV (°)
[18]	16	1	64
[19]	16	1	90
[20]	12	1	31.9
[34]	16	1	36.2
[35]	64	1	∼84
This work	8	2	133

## 7 Conclusions

This work presents the design and experimental demonstration of an 8-antenna dual-mode optical phased array receiving chip that leverages mode diversity to significantly extend the field of view. The integration of a low-loss mode splitter-converter enables efficient and flexible management of signals across different spatial modes. Furthermore, we incorporate an optical coherent combining structure to optimally utilize collected photons for coherent detection. This mechanism ensures efficient and coherent combination of signals from multiple channels, enhancing the overall receiving performance. Our proof-of-concept demonstrates the substantial advantages of employing higher-order spatial modes in OPA receivers. The successful integration of mode diversity and coherent combination within a single chip holds immense promise for scaling OPA technology in LiDAR and FSO applications. These techniques not only enhance FOV and signal processing capabilities but also provide new avenues for optimizing and expanding OPA systems, leading to increased reliability and adaptability in diverse operational scenarios.

## References

[1] Hsu C.-P. (2021). A review and perspective on optical phased array for automotive LiDAR. *IEEE J. Sel. Top. Quantum Electron.*.

[2] Wu Y. (2022). Multi-beam optical phase array for long-range LiDAR and free-space data communication. *Opt Laser. Technol.*.

[3] Poulton C. V. (2019). Long-range LiDAR and free-space data communication with high-performance optical phased arrays. *IEEE J. Sel. Top. Quantum Electron.*.

[4] Liu Y., Hu H. (2022). Silicon optical phased array with a 180-degree field of view for 2D optical beam steering. *Optica*.

[5] Li N. (2022). A progress review on solid-state LiDAR and nanophotonics-based LiDAR sensors. *Laser Photon. Rev.*.

[6] Zhang M. (2023). Phase-modulated continuous-wave coherent ranging method for optical phased array lidar. *Opt. Exp.*.

[7] Hu H. (2024). Silicon-based optical phased array with a reconfigurable aperture for “gaze” scanning of LiDAR. *Photon. Res.*.

[8] Poulton C. V. (2022). Coherent LiDAR with an 8,192-element optical phased array and driving laser. *IEEE J. Sel. Top. Quantum Electron.*.

[9] Miller S. A. (2020). Large-scale optical phased array using a low-power multi-pass silicon photonic platform. *Optica*.

[10] Chung S., Abediasl H., Hashemi H. (2018). A monolithically integrated large-scale optical phased array in silicon-on-insulator CMOS. *IEEE J. Solid-State Circ.*.

[11] Sun C. (2022). Large-scale and broadband silicon nitride optical phased arrays. *IEEE J. Sel. Top. Quantum Electron.*.

[12] Xu W., Zhou L., Lu L., Chen J. (2019). Aliasing-free optical phased array beam-steering with a plateau envelope. *Opt. Express*.

[13] Yan X., Chen J., Dai D., Shi Y. (2021). Polarization multiplexing silicon-photonic optical phased array for 2D wide-angle optical beam steering. *IEEE Photonics J.*.

[14] Muñoz P. (2022). Scalable switched slab coupler based optical phased array on silicon nitride. *IEEE J. Sel. Top. Quantum Electron.*.

[15] Hashemi H. (2021). A review of semiconductor-based monolithic optical phased array architectures. *IEEE Open J. Solid-State Circ. Society*.

[16] Shim J. (2020). On-chip monitoring of far-field patterns using a planar diffractor in a silicon-based optical phased array. *Opt. Lett.*.

[17] Zhang L.-X. (2020). Large-scale integrated multi-lines optical phased array chip. *IEEE Photonics J.*.

[18] Kossey M. R., Rizk C., Foster A. C. (2018). End-fire silicon optical phased array with half-wavelength spacing. *APL Photonics*.

[19] Liang D. (2023). Grating lobe-free silicon optical phased array with periodically bending modulation of dense antennas. *Opt. Express*.

[20] Kwong D., Hosseini A., Zhang Y., Chen R. T. (2011). 1×12 Unequally spaced waveguide array for actively tuned optical phased array on a silicon nanomembrane. *Appl. Phys. Lett.*.

[21] Badal M. T. I., Scott J., Wang K. (2023). Multimode optical phased array for parallel beam steering – feasibility study. *Opt. Express*.

[22] Fatemi R., Khial P. P., Khachaturian A., Hajimiri A. (2021). Breaking FOV-aperture trade-off with multimode nano-photonic antennas. *IEEE J. Sel. Top. Quantum Electron.*.

[23] Qiu H., Liu Y., Meng X., Guan X., Ding Y., Hu H. (2024). Ultra-sparse aperiodic silicon optical phased array using high-performance thermo-optic phase shifter. *Laser Photonics Rev.*.

[24] Sun C., Yu Y., Ye M., Chen G., Zhang X. (2016). An ultra-low crosstalk and broadband two-mode (de)multiplexer based on adiabatic couplers. *Sci. Rep.*.

[25] Priti R. B., Liboiron-Ladouceur O. (2018). A reconfigurable multimode demultiplexer/switch for mode-multiplexed silicon Photonics interconnects. *IEEE J. Sel. Top. Quantum Electron.*.

[26] Paredes B., Mohammed Z., Villegas J., Rasras M. (2021). Dual-band (O & C-bands) two-mode multiplexer on the SOI platform. *IEEE Photonics J.*.

[27] Jiang W., Mao S., Hu J. (2024). Ultra-compact silicon mode (de) multiplexer using inverse-designed adiabatic coupler. *J. Lightwave Technol.*.

[28] Zhang E., Yang S., Zhang L. (2024). General waveguide bend design based on cubic spline interpolation and inverse design. *J. Lightwave Technol.*.

[29] Miller D. A. B. (2013). Self-aligning universal beam coupler. *Opt. Express*.

[30] Bogaerts W. (2020). Programmable photonic circuits. *Nature*.

[31] Cao X., Zheng S., Long Y., Ruan Z., Luo Y., Wang J. (2020). Mesh-structure-enabled programmable multitask photonic signal processor on a silicon chip. *ACS Photonics*.

[32] Roques-Carmes C., Fan S., Miller D. A. B. (2024). Measuring, processing, and generating partially coherent light with self-configuring optics. *Light Sci. Appl.*.

[33] SeyedinNavadeh S. (2024). Determining the optimal communication channels of arbitrary optical systems using integrated photonic processors. *Nat. Photonics*.

[34] Li W., Chen J., Liang D., Dai D., Shi Y. (2022). Silicon optical phased array with calibration-free phase shifters. *Opt. Express*.

[35] Phare C. T. (2018). Silicon optical phased array with high-efficiency beam formation over 180 degree field of view. *arXiv:1802.04624*.

